# Delivery at Six Months with Membranes Unruptured

**Published:** 1882-08

**Authors:** P. W. Van Peyma


### Delivery at Six Months with Membranes Unruptured.

I was called during the night of July 17th, to attend Mrs. B., in labor for the fourth time; previous parturitions normal. On arriving at the house and making an examination, I discovered protruding from the vulva a smooth amniotic sac containing the foetus. Upon applying slight traction by means of a towel wrapped around the mass, it was easily removed. The uterus immediately contracted firmly and so continued. Upon examination of the sac and contents, it was found to be the entire unruptured amniotic sac containing the dead foetus as well as placenta, &c. The contents were easily recognized on account of the transluscency of the membranes. The mother's last menstruation occurred more than six months previous, and the foetus had the appearance of having passed that age. Although similar cases have been reported, yet the literature upon the subject is exceedingly scanty and the cases very rare.

